# Co-treatment of Waste From Steelmaking Processes: Steel Slag-Based Carbon Capture and Storage by Mineralization

**DOI:** 10.3389/fchem.2020.571504

**Published:** 2020-10-16

**Authors:** Qing Zhao, Xinyi Chu, Xiaohui Mei, Qingzhang Meng, Jingyu Li, Chengjun Liu, Henrik Saxén, Ron Zevenhoven

**Affiliations:** ^1^Key Laboratory for Ecological Metallurgy of Multimetallic Mineral (Ministry of Education), Northeastern University, Shenyang, China; ^2^School of Metallurgy, Northeastern University, Shenyang, China; ^3^Process and Systems Engineering Laboratory, Åbo Akademi University, Åbo/Turku, Finland

**Keywords:** carbon capture and storage, steel slag, carbon emission reduction, waste management, mineralization

## Abstract

The iron and steel industry is an energy-intensive sector, and large amounts of waste/ by-products are generated during the steelmaking process, such as CO_2_, metallurgical slag, and wastewater. Enhancing the development and deployment of treating waste from the steelmaking process will be environment friendly and resource-saving. Capturing CO_2_ by steel slag (SS) via mineralization is regarded to be an excellent choice due to the high basicity of the slag. In this paper, recent research on the steel slag-based carbon capture and storage (SS-CCS) by mineralization was summarized. Three routes of SS-CCS are compared including, direct gas-solid carbonation, direct aqueous carbonation, and indirect carbonation, respectively. Furthermore, the challenges and prospects for further development of the SS-CCS were discussed.

## Introduction

As climate change progresses, the increasing frequency of natural disasters is causing more people to pay attention (Cai et al., 2017; Han et al., 2017; Zou et al., 2017; Shan et al., 2018; Yi et al., 2019). It is reasonable to believe that the contribution of CO_2_ emissions to climate change is 76% (Intergovernmental panel on climate change, 2014). The BP Statistical Review of World Energy published data of CO_2_ discharge from global energy consumption in 2018. Results indicated that the carbon emission of the world reached 33.89 Gt CO_2_, the fastest growth since 2010 of a total of 2.0% (British Petroleum, 2019). There is no doubt that industries and society have a duty-bound responsibility to reduce CO_2_ emissions as the current pace of progress is inconsistent with Paris's climate goals.

Anthropogenic CO_2_ emissions result primarily from the burning of carbon-based fossil fuels, and China, the United States, and India together account for more than two-thirds of global energy growth (British Petroleum, 2019; Cui et al., 2019). A large amount of fossil fuels are consumed which emit large volumes of CO_2_ from iron and steelmaking processes. The contribution of CO_2_ emissions from iron and steelmaking processes to global CO_2_ emissions is about 6–7% (Doucet, 2010). Furthermore, besides the CO_2_ emissions, a substantial amount of metallurgical slag is generated during iron and steelmaking processes. Steelmaking slag (SS) is mainly used in road construction and cement industries in many countries due to its similar composition to Portland cement (Gedam et al., 2012; Saly et al., 2018). However, some SSs can release large amounts of toxic metals (e.g., Cr) affecting their utilization (Huiting and Forssberg, 2003). Moreover, undesired alterations of minerals in SS could cause volume instability as a result of swelling and cracking phenomena, such as the hydration of alkaline earth metal oxides (CaO and MgO), and the transformation from α-C_2_S to γ-C_2_S (Shi, 2004; Wang et al., 2010; Yildirim and Prezzi, 2011). Researchers proposed that unstable CaO, MgO, and silicates can be used to capture and bind CO_2_ by mineral carbonation to reduce carbon emissions and create a (thermodynamically) more stable material (Huijgen and Comans, 2006; Wang et al., 2019).

## SS-CCS

SS can be classified into many types according to the steps of the smelting process, including electric arc furnace slag (EAFS), basic oxygen furnace slag (BOFS), ladle furnace slag (LFS), argon oxygen decarburization slag (AODS), and continuous casting slag (CCS). The methods of steel slag-based carbon capture and storage (SS–CCS) may be divided into a direct carbonation route and an indirect carbonation route (Ibrahim et al., 2019).

### Direct Carbonation

Direct SS carbonation, that is, the reaction of SS and CO_2_ accomplished in a single reaction step, includes two-phase (direct gas-solid carbonation) and three-phases (direct aqueous carbonation) routes (Huijgen and Comans, 2005; Librandi et al., 2019). Since the year 2000, researchers have completely a number of studies on the possibility and potential of SS's carbonation for the storage of CO_2_. Table 1 lists the reported carbonation efficiency for EAFS, BOFS, and LFS with different operating parameters. It can be seen in Table 1 that the optimum conditions obtained in various studies are highly dependent on the physical and chemical properties of SS (e.g., particle size, chemical composition, and mineralogy) (Polettini et al., 2016a). Gas-solid carbonation of SS suffers from rather long reaction rates as a result of the formation of a carbonate layer and calcium-depleted silicate zones, which hinder the diffusion of gas into the particle center (Butt et al., 1996). Pretreatments of SS, like grinding (CO_2_ uptake rate of SS is more than 80% wt. when the particle size is <0.08 mm) and thermal activation (830–850°C at 1 atm CO_2_ of BOFS) can improve the reaction rate and carbon capture rate, but are energy-intensive (Ron and Jens, 2002; Santos et al., 2012).

**Table 1:** Carbonation efficiency of different kinds of SS operating under various conditions.

**Material type**	**Carbonation conditions**					**Efficiency**	**References**
	**Method**	**Reactor**	**Solid comp**.	**Gas/liquid phases**	**Operating conditions**		
EAFS	Slurry carbonation	Stirred reactor	CaO:33.19% MgO:9.43% (d < 24.0 μm)	18.2% vol.CO_2_, 4.0% O_2_ 77.8% N_2_	Ambient temperature *t* = 10 min L/S = 10 (mL/g) *P* = 10.68 bar	52 g CO_2_/kg slag	Ghacham et al., 2016
BOFS	Aqueous carbonation	Stainless steel reactor	Ca:31% Mg:7.5% (*d* = 63–100 μm)	40% vol. CO_2_ (*P* = 5 bar)	*T* = 50°C; *t* = 4 h; L/S = 5 (mL/g)	53.6 g CO_2_/100g slag	Polettini et al., 2016b
EAFS	Aqueous carbonation	Autoclave reactor	CaO:32.8% MgO:10% (d = 38–106 μm)	15% vol CO_2_, 85% vol N_2_	*T* = 20°C; *t* = 72 h L/S = 10 (mL/g) Q_L_ = 5 mL/min	1.47g CO_2_/100g slag	Danielle et al., 2008
LFS			CaO:58.1% MgO:6.2% (d < 38–106 μm)			24.7g CO_2_/100g slag	
BOFS	Aqueous carbonation	Rotating Packed Bed (RPB)	CaO:42.43% MgO:9.15% (d < 62 μm)	100% vol. CO_2_ (1.5kg/cm^2^)	*T* = 60°C; *t* = 30 min L/S = 20 (mL/g) Q_L_ = 1.2 L/min QCO_2_ = 2.5L/min	289 g CO_2_/Kg slag	Chang et al., 2012
EAFS	Accelerated carbonation	_	CaO = 29.9% MgO = 6.3% (d < 150 μm)	100% vol. CO_2_ (10 MPa)	L/S = 5 L/kg. *T* = 100°C *t* = 24 h	280 g CO_2_/kg slag	Baciocchi et al., 2015
EAFS	Gas-solid carbonation	Stainless steel batch reactor	CaO:28.27% MgO:7.88% (d < 2 mm)	99.99% vol. CO_2_	*T* = 30 ± 2°C; *t* = 3 h CO_2_ pressure=(3 bar)	11.1 g CO_2_/Kg slag	Rushendra et al., 2016
	Aqueous carbonation				*T* = 30 ± 2°C; L/S = 10 (mL/g) *t* = 3 h *P* = 6 bar	82 g CO_2_/Kg slag	

*BOFS, Basic oxygen furnace slags; EAFS, electric arc furnace slags; LFS, ladle furnace slag*.

Flue gases typically carry a lot of heat, and therefore direct gas-solid carbonation of SS for *in situ* CO_2_ sequestration is a promising process that recovers heat and increases the carbonation reaction rate. Tian et al. (2013) investigated the carbonation of SS with flue gas under various operational conditions. Temperature, CO_2_ concentration, and the presence of SO_2_ affect the carbon capture rate of the gas-solid carbonation of SS. The presence of SO_2_ has a positive effect on the SS mineralized CO_2_, especially during the diffusion-controlled stage, the reason probably being that SO_2_ increases the surface activation energy of SS, making CO_2_ easier to absorb. Though the maximum CO_2_ sequestration obtained at 151 ppm of SO_2_ was 88.5kg CO_2_ ${\text{t}}_{\text{slag}}^{-1}$ (76.1 kg CO_2_ ${\text{t}}_{\text{slag}}^{-1}$ without SO_2_ presence) which was, therefore, affected by the gas diffusion limitation.

Compared to the direct gas-solid carbonation of SS, a direct aqueous carbonation reaction has a higher chemical conversion rate. The three steps that occur in the reaction of SS with CO_2_ in aqueous solutions involve CO_2_ dissolution in water, Ca/Mg release from SS, and Ca or Mg carbonate precipitation. From the reaction mechanism of SS and CO_2_ in aqueous solution, it was found that the conversion rate of SS is related to the pressure of CO_2_, water amount, the amount of alkaline earth metal eluted from the SS, and the concentration of the lixiviant used. Pan et al. (2012) described the effect of CO_2_ pressure on the carbonation process, showing that an elevated pressure of 6 bar favors the dissolution of CO_2_ into the slurry (L/S, i.e., liquid to solid ratio, = 10 mL·g^−1^) at room temperature (Rushendra et al., 2016). However, there is an optimal CO_2_ pressure (CO_2_ pressure of 3 bar and L/S to 0.4 mL·g^−1^ at room temperature) on maximum CO_2_ uptake (13% wt.), beyond which the dissolution of SS becomes the limiting step of the carbonation process (Baciocchi et al., 2009). Beyond the optimal value, the carbonation efficiency (actual sequestration/theoretical sequestration capacity) of SS (27%) will not notably increase with the pressure but requires more heat and power (Rushendra et al., 2016). It was reported that a porous layer is formed during the Ca/Mg extraction from SS, which can obstruct further dissolution (Safari et al., 2009; Said et al., 2015). Furthermore, the reacted SS is covered by CaCO_3_ products, hindering gas diffusion and as a result also the carbonation reaction (Walton et al., 1997). It was observed that the thickness of the carbonated shell of the BOFS is about 200 μm, obtained at 200°C, 40% CO_2_ content, and 60% relative humidity (RH) (Ko et al., 2015). It is generally accepted that removing the “barrier layer” during the reaction is crucial to increase the dissolution rate of Ca/Mg from SS and to obtain high levels of conversion.

Researchers investigated the effects of operating conditions, such as temperature (25–100°C), particle size (<2 mm), and L/S (5–20 mL·g^−1^) on the kinetics of reactions (c.f., Table 1). It is necessary to closely control the temperature and water amount during the process of SS direct aqueous carbonation since increased temperatures can increase the carbonation reaction rate but reduce the solubility of CO_2_ in water (Bauer et al., 2011; Ukwattage et al., 2015). An appropriate water amount (L/S to 0.4 mL·g^−1^) can promote metal cations leaching from the SS (Baciocchi et al., 2010), while the presence of excessive water would block the diffusion of CO_2_ in the slurry (Fernández Bertos et al., 2004; Ko et al., 2015). Therefore, there has been an extensive research effort addressing the optimization of the liquid to solid ratio in the process, as low L/S ratios could reduce energy input requirements (differences in slag composition leads to different optimal L/S) (Sanna et al., 2014). Moreover, besides optimizing process parameters, changing the design of the reactor may promote carbonation progress. Compared to autoclave and slurry reactors, a rotating packed bed and rotary kiln is better dynamically, which can improve the contact probability between the gas and solid phases (Pan et al., 2014).

The carbonation process typically changes the original mineral composition of SS for several elements besides Ca and Mg. Mn silicate and oxide phases take part in the reaction and form new carbonates, causing a high dissolution degree of the original slag (Chang et al., 2018). There is a potential environmental risk that heavy metals and toxic elements, such as Cr, V, Ni, Pb, and Mo, elute during the carbonation process (Owais et al., 2019). Hence, Cr-bearing stainless steel slag (SSS) is rarely employed in the process of SS-CCS if the Cr exists in unstable phases. The authors of this paper studied the effects of chemical composition and cooling conditions on the crystallization behavior of stainless steel slag (Cao et al., 2018; Zhao et al., 2018a,b). It was proposed that a stabilization control of Cr during co-extraction of the Ca/Mg of SSS could be achieved by a modification treatment (Zhao et al., 2019a,b).

### Indirect Carbonation

The product layer produced during the direct SS mineralization of CO_2_ hinders the leaching of Ca/Mg from the SS and the diffusion of gas to the surface of unreacted phases. The route of indirect SS carbonation is divided into two steps: Ca/Mg is extracted from the SS before the carbonation process, with the two steps possibly occurring under different process conditions and concentrations (Sanna et al., 2015). The first step is the recovery of Ca/Mg from the SS by leaching, and the second step is the dissolution of CO_2_ in the leachate followed by carbonate precipitation. It performs best when the extraction and carbonation process are optimized separately. The operating parameters such as the particle size (<250 μm), chemical composition of SS (Mainly CaO and MgO content), type and concentration of additives (lixiviants) (acid, CH_3_COOH, H_2_SO_4_, HNO_3_, HCl and HCOOH, ammonium salt, NH_4_Cl, NH_4_NO_3_, and CH_3_COONH_4_), residence time (1–700 h), and solution temperature (25–100°C) were studied to improve the extraction yield of Ca/Mg (Mattila and Zevenhoven, 2014; Bilen et al., 2016; Wang et al., 2018). The carbonate precipitation rate mainly depends on the CO_2_ flow speed (0.5–2 L/min), size of gas bubbles (~1.2–10 mm), and the concentration of hydrogen ion in the solution, i.e., pH (Mattila et al., 2012; Xu et al., 2014).

Both weak and strong acids (H_2_SO_4_, HCl, HNO_3_, HCOOH, and CH_3_COOH) can selectively leach Ca/Mg from natural rock (i.e., serpentinite, magnesite, wollastonite) and alkaline solid waste from industrial activities (i.e., steel slag), to obtain a Ca/Mg salt solution (Teir et al., 2007, 2009). Selectively extracting Ca/Mg from SS is challenging to implement, some Fe, Si, or other elements may be eluted during the leaching process. Alkaline substances may be required to add to the leachate to recover Fe and promote the carbonation. It has been calculated that capturing and storing one ton of CO_2_ may require several tons of make-up chemicals (1842 kg CO_2_/t NaOH), which is costly and gives a high energy penalty (per ton of NaOH must consume 2,230 kWh of electricity and 0.44 tons of steam) (Wang et al., 2018). Also, often the reactor needs to be made of corrosion-resistant materials.

**Figure 1:**
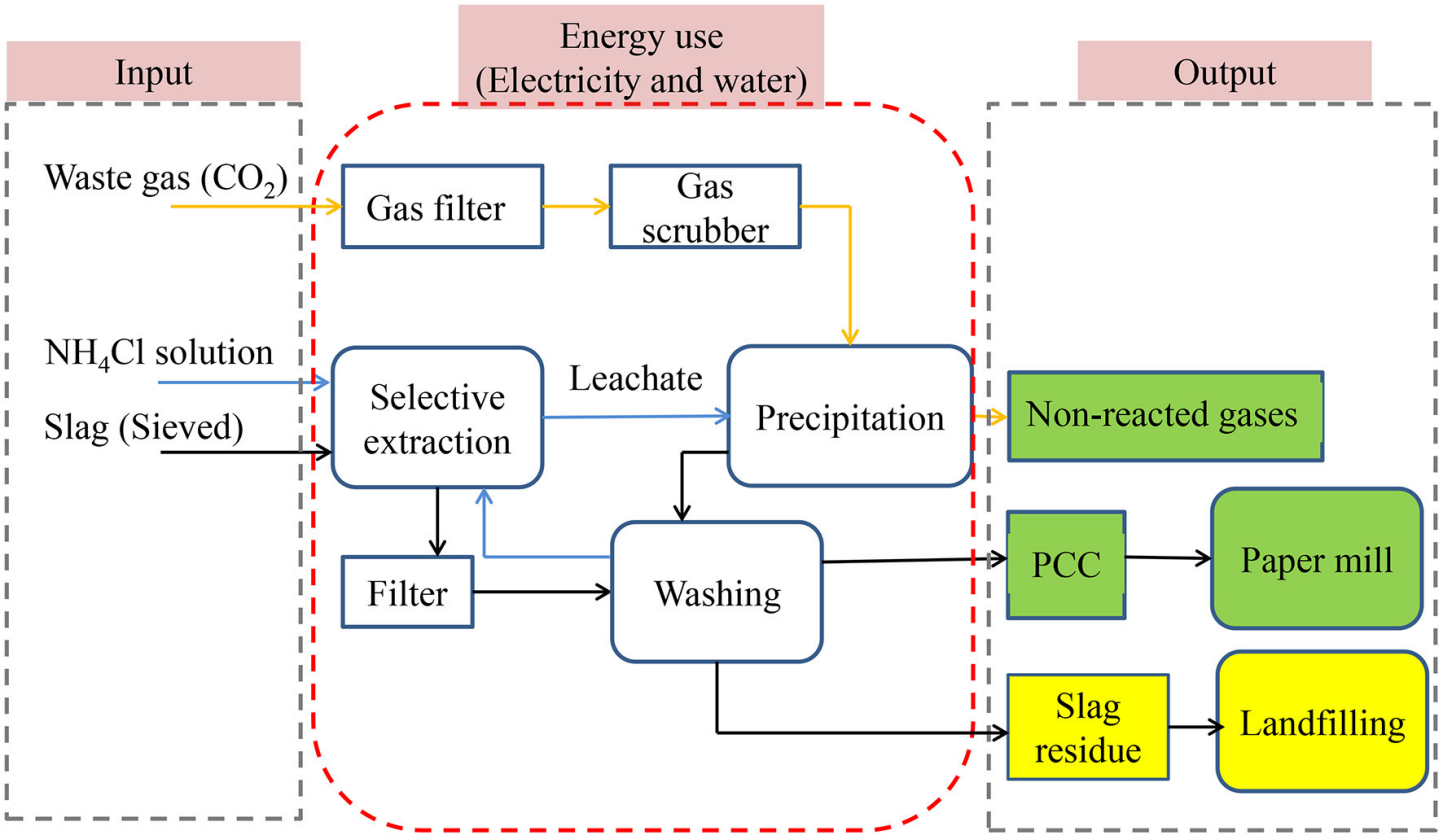
The slag2PCC route for PCC manufacturing from steelmaking slags: gas streams are in yellow arrows, aqueous streams are in blue arrows, and solid streams are in black arrows. Output items that are beneficial to the environment and resources are green, and items that have potential effects on the environment and resources are yellow.

Earlier work by Kodama et al. (2008); Teir (2008), and Eloneva et al. (2010a,b), Said et al. (2013) proposed that the aqueous solution of ammonium salt (NH_4_Cl, NH_4_NO_3_, and CH_3_COONH_4_) could be used to extract calcium from SS, and precipitated calcium carbonate (PCC) was prepared as a product. This route was named slag2PCC (c.f., Figure 1) by the team. The selective leaching of Ca from SS (c.f., Equation 1) and the formation of PCC (c.f., Equation 3) occur in the two separate extraction and carbonation reactors, respectively, while at the same time, residue slag and PCC products are removed via the circulation of the ammonium salt solvent solution between the extraction and carbonation reactors (Mattila and Zevenhoven, 2014; Said et al., 2016; Teir et al., 2016). Both the extraction and carbonation steps operate at ambient temperatures and pressures. The low temperature can suppress the ammonia slip and gives a lower energy penalty for solvent regeneration (Darde et al., 2009).

(1) $$\begin{array}{rcl} \text{CaO}\left(\text{s}\right)+\text{N}{\text{H}}_{4}\text{X}\left(\text{aq}\right) & + & {\text{H}}_{2}\text{O}\left(\text{l}\right)\to \text{Ca}{\text{X}}_{2}\left(\text{aq}\right)+2\text{N}{\text{H}}_{4}\text{OH}\left(\text{aq}\right)\text{X}\\ = & \text{C}{\text{l}}^{-1},\text{N}{\text{O}}_{3}^{-1},\text{C}{\text{H}}_{3}\text{CO}{\text{O}}^{-1} \end{array}$$

(2) $$\begin{array}{rcl} 2\text{N}{\text{H}}_{4}\text{OH}\left(\text{aq}\right) & + & \text{C}{\text{O}}_{2}\left(\text{g}\right)\to {\left(\text{N}{\text{H}}_{4}\right)}_{2}\text{C}{\text{O}}_{3}\left(\text{aq}\right)+{\text{H}}_{2}\text{O}\left(1\right) \end{array}$$

(3) $$\begin{array}{rcl} {\left(\text{N}{\text{H}}_{4}\right)}_{2}\text{C}{\text{O}}_{3}\left(\text{aq}\right) & + & \text{Ca}{\text{X}}_{2}\left(\text{aq}\right)\to \text{CaC}{\text{O}}_{3}\left(\text{s}\right)+2\text{N}{\text{H}}_{4}\text{X} \end{array}$$

The dissolution of CO_2_ in the aqueous ammonium salt solution is the speed-control part of the Slag2PCC process (c.f., Equation 2). Results reported that 4–5 mm CO_2_ bubbles dissolved when they rose ~1.6–1.9 m in an unmixed water column (Legendre and Zevenhoven, 2016, 2017). Recently, Zevenhoven et al. (2019) proposed that properly positioned mixing impellers can optimize the dissolution of CO_2_ bubbles at minimal mixing energy input. Incomplete CO_2_ dissolution is not only a flaw for the CCS but also a risk for an ammonia slip from the aqueous solution due to the dissolved ammonia possibly diffusing to and into CO_2_ bubbles (Zevenhoven et al., 2019). The second drawback is the ammonia evaporating from the solution and leaving with exit gas, which reduces the recycling of the ammonium salt solvent. Higher temperatures and lower solvent concentration can increase NH_3_ release (Said et al., 2016). Moreover, ammonium salt types also affect NH_3_ release (Eloneva et al., 2011). Furthermore, not all Ca be extracted from the slag, with about 70%-80% of Ca in converter slag (74–125 μm) dissolving in the ammonium nitrate solution (0.5–2 mol·L^−1^) (Mattila and Zevenhoven, 2014). Also a considerable amount of water may be needed to wash the PCC product and residue slag, which creates a significant environmental footprint (Hudd, 2014).

## Challenges

Although a large number of studies have focused on the reaction of CO_2_ with SS on a laboratory scale, it is still far from achieving the industrial level of several tons·h^−1^ processed. This is partly because of many challenges in terms of the differences in original mineralogy of SS, limitations of reaction kinetics, increase in energy and economic costs while optimizing process parameters, and minimizing environmental impacts.

### Phase Composition of Steel Slag

SS is a homogeneous solid waste and is composed of multiple mineral phases. Mineral phases such as bredigite (Ca_7_Mg(SiO_4_)_4_), srebrodolskite (Ca_2_Fe_2_O_5_), akermanite (Ca_2_MgSi_2_O_7_), β-and γ-polymorph (Ca_2_SiO_4_), merwinite (Ca_3_Mg(SiO_4_)_2_), and spinel (MgC_2_O_4_, mainly in stainless steel slag) exhibit varying degrees of carbonation kinetics during the process of mineralizing CO_2_. It has been reported that bredigite is the most reactive mineral, following by the polymorphs and wollastonite, while merwinite and diopside give the slowest carbonation conversions during a direct carbonation process (Bodor et al., 2013). The metallic oxide phases exhibited a relatively higher solubility than the silicate phases in aqueous solutions with various additives (Zhao et al., 2020). Therefore, non-sensitive Ca/Mg-bearing phases in SS reduce the CO_2_ capture rate and utilization rate of Ca and Mg resources. Furthermore, different types of SS, even the same kinds of SS from various steel mills, have different optimal process parameters for SS to capture CO_2_, because the main Ca/Mg-bearing phase of SS is different. Therefore, there are currently no systematic industrialization parameters suitable for the process of SS-CCS.

### Limitation of Reaction Kinetic

The formation of the product layer during the direct carbonation reaction is unavoidable and hinders the further chemical processing of metallurgical waste gas and SS. If physical or chemical methods can continuously remove the barrier layer during the reaction, the utilization rate of Ca/Mg resources in the SS can be significantly improved, and the emissions of unreacted CO_2_ gas can be reduced. Compared with the direct carbonation routes, the slag2PCC has a significantly higher chemical reaction rate, which can achieve selective leaching Ca and produce pure PCC. Not all CO_2_ gas molecules can be dissolved in this solution, and the dissolution of CO_2_ is the rate-controlling step of the slag2PCC process. NH_3_ loss from the solution lowers alkalinity and adds costs, which penalize the carbonation conversion and requires a subsequent gas treatment. Promoting the selective leaching rate of Ca in the steel slag and reaching higher efficiencies is necessary for the industrial-scale processing of the slag2PCC.

### Energy Input Requirements Environmental Impact

Both direct and indirect SS carbonation requires electricity for crushing, grinding, and sieving the slag aimed at increasing the reaction rate. It was found in an assessment that it takes 980–6,300 MJ t^−1^ CO_2_ and 246-427 kWh t^−1^ CO_2_ in the process of direct aqueous and indirect carbonation of SS, respectively, where the energy requirement is associated with heating, CO_2_ compression, and solid/liquid separation (Costa et al., 2016; Wang et al., 2018). Circulated pumping of the ammonium salt solution, gas compression input, solution agitation, and solids washing in the process of slag2PCC also requires power (Hudd, 2014). Besides the electricity, the washing of PCC products and recovery of solvent salts may require significant water as well, which increases the environmental burden. Therefore, it is necessary to systematically evaluate the relationship between energy input requirements and the benefit (and market value) of PCC products. Moreover, the treatment and application of carbonized slag and residual slag requires further study while the long-term leaching behavior of carbonated slag and residual slag and the potential environmental risks still need to be evaluated (Eloneva et al., 2010a; Zevenhoven et al., 2011).

## Prospects

The iron and steel industry is an energy-intensive sector, generating large amounts of waste during the steelmaking process, such as CO_2_, metallurgical slag, and wastewater. It is promising that it is possible to operate a CO_2_ capture process based on a reaction with SS on an industrial scale, since SSs are often cheap and readily available near large CO_2_ emitter sources. If physical or chemical methods can remove the barrier layer during the reaction, direct SS carbonation may be more natural to implement as a large-scale industrial application. This is not only due to the fact that the reaction is carried out in a single reactor, but also because the flue gas can provide all or part of the heat needed to improve the reaction's kinetics. In addition, modifying the mineral phase composition of the SS, which gives Ca/Mg in the sensitive phase of the carbonization reaction, has a positive effect on the application of SS–CCS.

Slag2PCC is a relatively complete process for co-processing steel slag and CO_2_ and is presently under investigation in pilot and laboratory scales (Said et al., 2013). In the future, it will be necessary to focus on avoiding NH_3_ losses from the solution and improving CO_2_ dissolution in the solutions for the slag2PCC process. Moreover, the energy input requirements of the slag2PCC process and the full economic and environmental benefits of the PCC still need to be systematically evaluated. The assessment results may differ for different countries and regions, which may be related to the scale of the steel mill and the distance between the steel mill and the application plant of PCC. Also, the treatment of solid residues generated by the SS-CCS process and environmental risk assessments need further study.

## Author Contributions

QZ carried out the concepts and design of the article. XC, QM, and JL provided literature research. XM carried out manuscript editing. CL, HS, and RZ performed manuscript review. All authors contributed to the article and approved the submitted version.

## Conflict of Interest

The authors declare that the research was conducted in the absence of any commercial or financial relationships that could be construed as a potential conflict of interest.
